# Training-free measures based on algorithmic probability identify high nucleosome occupancy in DNA sequences

**DOI:** 10.1093/nar/gkz750

**Published:** 2019-09-12

**Authors:** Hector Zenil, Peter Minary

**Affiliations:** 1 Oxford Immune Algorithmics, Oxford University Innovation, Oxford, UK; 2 Algorithmic Dynamics Lab, Unit of Computational Medicine, SciLifeLab, Center for Molecular Medicine, Karolinska Institute, Stockholm, Sweden; 3 Algorithmic Nature Group, LABORES for the Natural and Digital Sciences, Paris, France; 4 Department of Computer Science, University of Oxford, Oxford, UK

## Abstract

We introduce and study a set of training-free methods of an information-theoretic and algorithmic complexity nature that we apply to DNA sequences to identify their potential to identify nucleosomal binding sites. We test the measures on well-studied genomic sequences of different sizes drawn from different sources. The measures reveal the known *in vivo* versus *in vitro* predictive discrepancies and uncover their potential to pinpoint high and low nucleosome occupancy. We explore different possible signals within and beyond the nucleosome length and find that the complexity indices are informative of nucleosome occupancy. We found that, while it is clear that the gold standard Kaplan model is driven by GC content (by design) and by *k*-mer training; for high occupancy, entropy and complexity-based scores are also informative and can complement the Kaplan model.

## INTRODUCTION

DNA in the cell is organised into a compact form, called chromatin (1). One level of chromatin organisation consists in DNA wrapped around histone proteins, forming nucleosomes (2). A nucleosome is a basic unit of DNA packaging. Depending on the context, nucleosomes can inhibit or facilitate transcription factor binding and are thus a very active area of research. The location of low nucleosomal occupancy is key to understanding active regulatory elements and genetic regulation that is not directly encoded in the genome but rather in a structural layer of information.

The structural organisation of DNA in the chromosomes is widely known to be heavily driven by GC content (3), notwithstanding that *k*-mer approaches have been discovered to increase predictive power (4–6). Indeed, local and short-range signals carried by DNA sequence motifs or ‘fingertips’ have been found to be able to determine a good fraction of the structural (and thus functional) properties of DNA, such as nucleosome occupancy, with significant differences for *in vivo* versus *in vitro* data (7).

Intensive analysis of the statistical correspondence between DNA sequence and *in vitro* and *in vivo* positioning has shown the degree to which the nucleosome landscape is intrinsically specified by the DNA sequence (8). Here, we consider a set of algorithmic and information-theoretic complexity measures to help unveil how much of the information encoded in a sequence in the context of the nucleosome landscape can be recovered from training-free information-content and algorithmic-complexity measures, i.e. with no previous knowledge, such as informative *k*-mers. Nucleosome location is an ideal test case to probe how informative sequence-based indices of complexity can be in determining structural (and thus some functional) properties of genomic DNA, and how much these measures can both reveal and encode.

### Information-theoretic approaches to genomic profiling

Previous applications based upon algorithmic complexity include experiments on the evaluation of lossless compression lengths of sets of genomes (9,10), and more recently, in (11), demonstrating applications of algorithmic complexity to DNA sequences. In a landmark paper in the area, a measure of algorithmic mutual information was introduced to distinguish sequence similarities by way of minimal encodings and lossless compression algorithms in which a mitochondrial phylogenetic tree that conformed to the evolutionary history of known species was reconstructed (10,12).

However, most of these approaches have either been purely theoretical or have been effectively reduced to applications or variations of Shannon entropy (13) rather than of algorithmic complexity, because popular implementations of lossless compression algorithms are actually closer to Shannon entropy than to algorithmic complexity (14,15).

In certain cases, some control tests have been missing. For example, in the comparison of the similarity distances of different animal genomes (10,12) based on lossless compression, GC content (counting every G and C in the sequence) can reconstruct an animal phylogenetic tree as accurate as the one produced in (16). This is because two species that are close to each other evolutionarily will also have similar GC content.

Species close to each other will have similar DNA sequence entropy values, allowing lossless compression algorithms to compress statistical regularities of genomes of related species with similar compression rates. Indeed, the GC content of every species can be mapped to a single point on a Bernoulli-shaped curve typically used to illustrate the distribution of Shannon entropy across a set of strings, in this case a set of genome sequences with entropy corresponding to the count of G or C versus A or T. The result will be that two species have similar genomic entropy if they have similar GC content. Here, we intend to go beyond this—in breadth as well as depth–using better-grounded algorithmic measures and more biologically relevant test cases.

### Current sequence-based prediction methods

While the calculation of GC content is extremely simple, the reasons behind its ability to predict the structural properties of DNA are not completely understood (3,17). For example, it has been shown that low GC content can explain low occupancy, but high GC content can mean either high or low occupancy (18) and this is why the methods here introduced may prove to be highly valuable. But how much GC content alone encodes nucleosome position, given that DNA (and thus GC content) encodes much more than chromatin structure, is a topic of interest. The same DNA sequences are constrained within functional/evolutionary trajectories, such as protein coding versus non-coding and regulatory versus non regulatory trajectories, among others. The *in vitro* and *in vivo* discrepancy can be explained in the same terms, with other factors such as chromatin remodellers and transcription factors affecting nucleosome organisation differently *in vitro* versus *in vivo*.

Current algorithms that build upon, while probing beyond GC content, have been largely influenced by sequence motif (6,19) and dinucleotide models (20)—and to a lesser degree by *k*-mers (5)—and thus are not training- free, and are the result of years of experimental research.

### The dinucleotide wedge model

The formulation of models of DNA bending was initially prompted by a recognition that DNA must be bent for packaging into nucleosomes, and that bending would be an informative index of nucleosome occupancy. Various dinucleotide models can account reasonably well for the intrinsic bending observed in different sets of sequences, especially those containing A-tracts (21).

The *Wedge model* (22) suggests that bending is the result of driving a wedge between adjacent base pairs at various positions in the DNA. The model assumes that bending can be explained by wedge properties attributed solely to an AA dinucleotide (8.7 degrees for each AA). No current model provides a completely accurate explanation of the physical properties of DNA such as bending (23), but the Wedge model (like the more basic Junction model, which is less suitable for short sequences and less general (24)) reasonably predicts the bending of many DNA sequences (25). Although it has been suggested that trinucleotide models may make for greater accuracy in explaining DNA curvature in some sequences, dinucleotide models remain the most effective (21).

### The Kaplan model

Kaplan *et al.* established a probabilistic model to demonstrate the possibility that one DNA sequence may be occupied by a nucleosome (7). They constructed a nucleosome–DNA interaction model and used a hidden Markov model (HMM) to obtain a probability score. The model is based mainly on a 10-bp sequence periodicity that indicates the probability of any base pair being covered by a nucleosome. The Kaplan model is considered the most accurate, and is the gold standard for predicting in vitro nucleosome occupancy. However, previous approaches, including Segal’s (26) and Kaplan’s (7), require extensive (pre-)training. In contrast, all measures considered in our approach are training-free. The model of Kaplan *et al.* is considered the gold standard for comparison purposes.

## MATERIALS AND METHODS

To study the extent to which some signals contribute to the determination of nucleosome occupancy, we applied some basic transformations to the original genomic DNA sequence. The SW transformation substitutes G and C for S (Strong interaction), and A and T for W (Weak interaction). The RY transformation substitutes A and G for R (puRines) and C and T for Y (pYrimidines).

### Complexity-based genomic profiling

In what follows, we generate a function score *f*_c_ for every complexity measure *c* (detailed descriptions in the Sup. Mat.) by applying each measure to a sliding window of length 147 nucleotides (nts) across a 20K and 100K base pair (bp) DNA sequence from Yeast chromosome 14 (3). At every position of the sliding window, we get a function score for every complexity index *c* applied to the sequence of interest used to compare *in vivo* and *in vitro* occupancies.

The following measures are introduced. First those classical and of wider use (see Supplementary Material for definitions):

- Shannon entropy with uniform probability distribution.
- Entropy rate with uniform probability distribution.
- Lossless compression (based on the Lempel-Ziv-Welch algorithmic or LZW)

And a set of measures based or motivated in algorithmic complexity (see Supplementary Material for exact definitions):

- Coding Theorem Method (CTM) as an estimator of algorithmic randomness by way of algorithmic probability via the algorithmic Coding theorem (see Supplementary Material) relating causal content and classical probability (27,28).
- Logical depth (LD) as a BDM-based (see below) estimation of logical depth (29), a measure of sophistication that assigns both algorithmically simple and algorithmically random sequences shallow depth, and everything else higher complexity, believed to be related to biological evolution (30,31).

And a hybrid measure of complexity combining local approximations of algorithmic complexity by CTM and global estimations of (block) Shannon entropy (see Supplementary Material for exact definitions):

- The Block Decomposition Method (BDM) that approximates Shannon entropy—up to a logarithmic term—for long sequences, but Kolmogorov–Chaitin complexity (32,33) otherwise, as in the case of short nucleotides (34).

We list lossless compression under information-theoretic measures and not under algorithmic complexity measures, because popular implementations of lossless compression algorithms such as Compress and all those based on Lempel-Ziv-Welch (LZ or LZW), as well as derived algorithms (ZIP, GZIP, PNG, etc.), are actually entropy estimators (14,15,34).

BDM allows us to expand the range of application of both CTM and LD to longer sequences by using Shannon entropy. However, if sequences are divided into short-enough subsequences (of 12 nucleotides), we can apply CTM and avoid any trivial connection to Shannon entropy, and thus to GC content.

Briefly, to estimate the *algorithmic probability* (35,36)—on which the measure BDM is based—of a DNA sequence (e.g. the sliding window of length 147 nucleoides or nt), we produce an empirical distribution (27,28) to compare with by running a sample of 2.5 × 10^13^ Turing machines with two states and five symbols (which is also the number of nucleotide types in a DNA sequence) with empty input. If a DNA sequence is algorithmically random, then very few computer programs (Turing machines) will produce it, but if it has a regularity, either statistical or algorithmic, then there is a high probability of it being produced. Producing approximations to algorithmic probability provides approximations to algorithmic complexity by way of the so-called *algorithmic Coding Theorem* (36,27,28). Because the procedure is computationally expensive (and ultimately uncomputable), only the full set of strings of up to 12 bits was produced, and thus direct values can be given only to DNA sequences of up to 12 digits (binary for RY and SW and quaternary for full-alphabet DNA sequences).

The same methods (CTM and BDM) that we will use along the paper are also used in the context of applications of data deconvolution and network biology (37–39). A review of some of these measures is available in (40).

## RESULTS

Table 1 shows the *in vitro* nucleosome occupancy dependence on GC content, with a correlation of 0.684 (similar to that reported by Kaplan (7)) for the well-studied 20K bp genomic region (187K–207K) of Yeast Chromosome 14, exactly as was reported in (26) using their data, with no sliding window but on full sequences. Knowledge-based methods dependent on observed sequence motifs (41) are computationally cost-effective alternatives for predicting genome-wide nucleosome occupancy. However, they are trained on experimental statistical data and are not able to predict anything that has not been observed before. They also require context, as it may not be sufficient to consider only short sequence motifs, such as dinucleotides (7,21).

**Table 1.** Spearman correlations between complexity indices with *in vivo* and *in vitro* experimental nucleosome occupancy data from position 187 001 bp to 207 000 bp on the 14th yeast chromosome

	*In vitro*	*In vivo*
*In vitro*	1	0.5
*In vivo*	0.5	1
GC content	0.684	0.26
LD	−0.29	−0.23
Entropy	0.588	0.291
BDM	0.483	0.322
Compress	0.215	0.178

### Complexity-based indices

**Figure 1.**
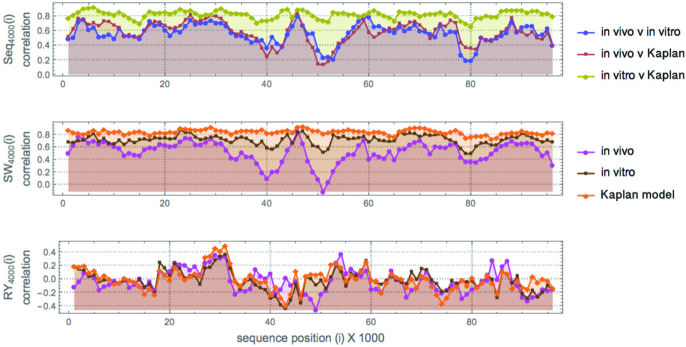
Top: Scaled score values of nucleosome occupancy in the 14th Yeast chromosome (experimentally validated versus the Kaplan model) on a sliding window of length 4K nt for both in vitro and *in vivo* data against different measures/signals: the occupancy predicting Kaplan model (clearly better for in vitro). Middle: SW is simply GC written as SW in contrast to RY (which is not AT). Calculated correlation values are highly correlated to the Kaplan model but are poor at explaining *in vivo* occupancy data. Bottom: The RY DNA transformation, a signal orthogonal to SW (and thus to GC content), whose values report a non-negligible max-min correlation, suggesting that the mixing of AT and GC carries some information about nucleosome occupancy (even if weaker than GC content), with *in vivo* values showing the greatest correlation, unlike SW/GC, thus possibly neglected in predictive models (such as Kaplan’s). The starting and ending points of the 100K segment are 187K − 40K and 207K + 40K nts in the 14th Yeast chromosome surrounding the 20K sequence studied in (3,26).

Figure 1 shows the correlations between *in vivo, in vitro* data and the Kaplan model. In contrast, the SW transformation captures GC content, which clearly drives most of the nucleosome occupancy, but the correlation with the RY transformation that loses all GC content is very interesting. While significantly lower, it does exist, and indicates a signal not contained in the GC content alone, as verified in Figure 4.

In Table 1, we report the correlation values found between experimental nucleosome occupancy data and ab initio training-free complexity measures. BDM alone explains more than any other index, including GC content *in vivo*, and unlike all other measures LD is negatively correlated, as theoretically expected (42) and numerically achieved (34), it being a measure that assigns low logical depth to high algorithmic randomness, with high algorithmic randomness implying high entropy (but not the converse).

Entropy alone does not capture all the GC signals, which means that there is more structure in the distributions of Gs and Cs beyond the GC content alone. However, entropy does capture GC content *in vivo*, suggesting that local nucleotide arrangements (for example, sequence motifs) have a greater impact on *in vivo* prediction. Compared to entropy, BDM displays a higher correlation with *in vivo* nucleosome occupancy, thereby suggesting more internal structure than is captured by GC content and entropy alone, that is, sequence structure that displays no statistical regularities but is possibly algorithmic in nature.

### Model curvature versus complexity indices

The dinucleotide model incorporates knowledge regarding sequence motifs that are known to have specific natural curvature properties, and adds to the knowledge and predictive power that GC content alone offers.

Using the Wedge dinucleotide model we first estimated the predicted curvature on a set of 20 artificially generated sequences (Supplementary Table S3 (Supplementary. Material)) with different statistical properties, in order to identify possibly informative information-theoretic and algorithmic indices. As shown in Supplementary Table S1 (Supplementary. Material), we found all measures negatively correlated to the curvature modelled, except for LD, which displays a positive correlation—and the highest in absolute value—compared to all the others. This is consonant with the theoretically predicted relation between algorithmic complexity (CTM and BDM) and logical depth (LD) (34). All other measures (except for LD) behave similarly to BDM. Since BDM negatively correlates with curvature, it is expected that the minima may identify nucleosome positions (see next subsection).

The results in Table 1 and Supplementary Table S1 (Sup. Mat.) imply that for all measures, extrema values may be indicative of high nucleosome occupancy. In the next section we explore whether extrema of these measures are also informative about nucleosome location.

According to Table 1 there is a positive correlation between nucleosome occupancy and therefore one would expect to see higher nucleosome occupancy at higher values of BDM. This only result by itself would imply that the maximum of BDM would be more informative about nucleosome positions.

### Nucleosome dyad and centre location test

The positioning and occupancy of nucleosomes are closely related. Nucleosome positioning is the distribution of individual nucleosomes along the DNA sequence and can be described by the location of a single reference point on the nucleosome, such as its dyad of symmetry (43). Nucleosome occupancy, on the other hand, is a measure of the probability that a certain DNA region is wrapped around a histone octamer.

Here, we have taken a set of sequences that are, to our knowledge, among the most studied in the context of nucleosome research. Their structural properties have been experimentally validated, making them ideal for testing any measure on, and they have also been used in other studies.

**Figure 2.**
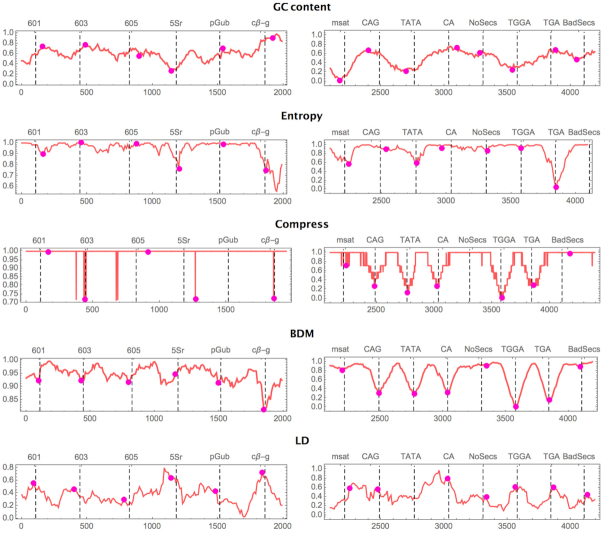
Nucleosome centre location according to five indices on 14 much-studied and experimentally validated nucleosomes in Yeast (source and full sequences listed in Table S5) intercalated with another 16 pseudo-random DNA segments of 147 nts with the same average GC content as one of the immediate neighbouring legitimate nucleosomal sequences to erase any GC content difference on purpose. Values are normalised between 0 and 1 and they were smoothed by taking one data point per 10. The *y* axis scales differ between the left and right panels for ease of illustration only. Experimentally known nucleosome centres (called dyads) are marked with dashed lines and the centres located, according to each measure, are marked with a magenta circle. Panels on the right for which no dyad is known have their centre estimated by the centre of the nucleosomal sequence. By design GC content performs poorly, but entropy recovers the signal ab initio. Centre predictions were called based on the local (147 nt window) minimum; only GC Content was called based on either the local minimum or local maximum (min/max), thus giving it an extra edge. LD centre calls were made to the local maximum. Values for the best performer index, BDM, are reported in Table 3. We know, however, that the Kaplan model relies heavily on GC-content and *K*-mers, so we expect that it would be fooled when flanked by sequences of similar GC content.

**Figure 3.**
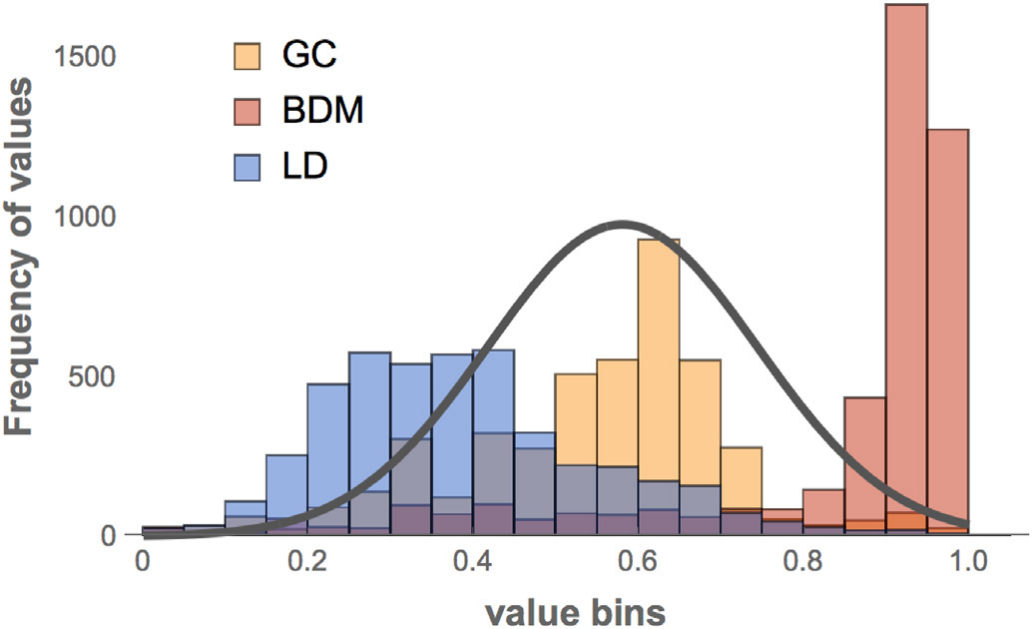
Histogram of values for each complexity score in Figure 2. On the *x*-axis are the complexity values arranged in bins of size 0.05 spanning 0 to 1 (the normalised complexity scores reported in Figure 2), while the *y*-axis shows how many times the same value repeats. The plot demonstrates how BDM and LD are the most removed from normal, unlike GC content. A normal probability distribution is plotted in black, with mean and std deviation estimated from the GC content values for comparison purposes. BDM carries the strongest signal, followed by LD skewed in the opposite direction, as expected, given its relationship to algorithmic complexity as estimated by BDM.

Figure 2 shows the location capabilities of algorithmic indices for nucleosome dyad and centre location when nucleosomal regions are placed against a background of (pseudo-) randomly generated DNA sequences with the same average GC content as the immediate left legitimate neighbour. As illustrated, BDM outperforms all methods in accuracy (Figure 2 and Supplementary Table S2 (Supplementary Material)) and in signal strength (Figure 3). The results indicate an emerging trend. For algorithmic and information-theoretic measures the minimum is correlated with nucleosomal centre location, except for LD (which is weakly negatively correlated). GC content fails even if we give it the advantage of taking both min and max values as possible indicators of the nucleosome centre. This is because we designed the experiment for GC content to fail by surrounding the nucleosome regions by pseudo-random DNA sequences with GC content similar to the surrounded nucleosomal regions. Yet the complexity-based indexes were still able to pinpoint the locations consistently and more accurately.

The results for BDM and LD suggest that the first four nucleosomal DNA sequences, of which three are clones, display greater algorithmic randomness (BDM) than the statistically pseudo-randomly generated background (surrounding each legitimate sequence) designed to erase any GC content difference, while all other nucleosomes are of significantly lower algorithmic randomness (BDM) and mixed (both high and low) structural complexity (LD). Structural complexity in the context of LD means sequences that are deep in computational content, that is, they are neither random nor trivial and they require computational work (the segments can only be generated by a slow computer program). The same robust results were obtained after several replications with different pseudo-random backgrounds. Moreover, the signal produced by similar nucleosomes with strong properties (44), such as clones 601, 603 and 605, had similar shapes and convexity. The results suggest that algorithmic and information-theoretic measures can recover a strong signal and can complement GC content and *K*-mer training in finding nucleosome positions and nucleosomal centres.

Figure 3 shows the strength of noise versus signal in the distribution of values for three indices (min values for BDM, max values for LD, and min/max values for GC content). The signal-to-noise ratio is much stronger for BDM and LD, but LD is shifted in the opposite direction (to BDM), consistent with the theoretical expectation (what is highly random for BDM is shallow for LD) (see Supplementary Material), but for GC content the distribution is normal, indicating that GC content distributes values no much better than random (which was expected by design), unlike BDM, that performs better even on exactly the same data. In other words, both BDM and LD spike at nucleosome positions stronger and removed from normality compared to GC content on nucleosomal regions on a pseudo-random DNA background (with GC content the same as each flanked nucleosomal region). BDM is informative about every dyad or centre of a nucleosome, with 8 out of the 14 predicted within 1 to 7 nts distance. Unlike all other measures, LD performed better for the first half (left panel 2) of nucleosome centre locations than for the second half (right panel 2), suggesting that the nucleosomes of the first half may have greater structural organisation. BDM outperforms all other indices.

Supplementary Table S2 (Supplementary Material) compares distances to the nucleosome centres as predicted without any training, with BDM outperforming GC content, as shown in Figure 2. The average distance between the predicted and the actual nucleosome centre is calculated to the closest local extreme (minima or maxima) for GC content and only minima for BDM (hence giving GC content an advantage) within a window of 73 bps from the actual centre (the experimentally known dyad, or the centre nucleotide when the dyad is not known).

In accordance with the results provided in Figure 2 and Table 1, the minima of BDM is informative for nucleosome position for the 14 test sequences whose natural curvature is a fit to the superhelix. The minima of BDM (maxima of LD) may thus also indicate nucleosome location. This latter finding is supported by results in Supplementary Table S2.

Our results suggest that if some measures of complexity indicate high or low occupancy nucleosomal regions where GC content fails, the measures may capture structural signals different from GC content, such as *k*-mers, accounting for <20% of the accuracy of the Kaplan model (with the rest owing to GC content alone). However, the strong signal captured by some complexity measures and the marks found in signals complementary to GC content (RY content) suggest that these complexity measures are not only able to capture the usual markers, such as GC content, with, e.g., Shannon entropy alone, but also *k*-mer knowledge, without any previous knowledge or training. Furthermore, the measures may be revealing signals complementary to GC content running along the DNA not revealed hitherto and requiring further examination.

### Informative measures of high and low occupancy

To find the most informative measures of complexity *c* we maximised the separation by taking only the sequences with the highest 2% and lowest 0.2% nucleosome occupancy from a 100K DNA segment for highest and lowest nucleosome occupancy values. There were 7701 high and 5649 low occupancy in vitro sequences, and 4332 high and 3989 low *in vivo* sequences. The starting and ending points of the 100K segment are 187K–40K and 207K + 40K nts in the same 14th Yeast chromosome (3,26), that is, 40K nts surrounding the original shorter 20K sequence first studied in this paper.

The box plot for the Kaplan model indicates that the model may not work as well for extreme sequences of high occupancy where the maximum over the segments on which these nucleosome regions are contained reaches an average correlation of ∼0.85 (in terms of occupancy), as shown in Figure 1 for *in vitro* data. This means that these high occupancy sequences may be on the outer border of the standard deviation in terms of accuracy in the Kaplan model.

The best model is the one that best separates the highest from the lowest occupancy, and therefore is clearly Kaplan’s model. Except for information-theoretic indices (Entropy and Compress), all algorithmic complexity indices were found to be informative of high and low occupancy. Moreover, all algorithmic complexity measures display a slight reduction in accuracy *in vivo* versus *in vitro*, as is consistent with the literature. All but the Kaplan model, however, are training-free measures, in the sense that they do not contain any prior *k*-mer bias related to high and low occupancy and thus are naive indices. Yet all algorithmic complexity measures were informative to different extents, with entropy, CTM and BDM performing best and LD performing worst, and LD displaying inverted values for high and low occupancy as theoretically expected (because LD assigns low LD to high algorithmic complexity) (42). Also of note is the fact that CTM and BDM applied to the RY transformation were informative of high versus low occupancy, thereby revealing a signal different from GC content that models such as Kaplan’s may only partially capture in their *k*-mer training.

Lossless compression was the worst behaved, showing how CTM and BDM outperform what is usually used as an estimator of algorithmic complexity (14,15,34). Unlike entropy alone, however, lossless compression does take into consideration sequence repetitions, averaging over all *k*-mers up to the compression algorithm sliding window length. The results thus indicate that averaging over all sequence motifs—both informative and not—deletes all advantages, thereby justifying specific knowledge-driven *k*-mer approaches introduced in models such as Segal’s and Kaplan’s.

## CONCLUSIONS

Current gold standard prediction methods for nucleosome location correlate highly with GC content and require extensive (pre-)training to refine what GC content can achieve.

More recently, deep machine learning techniques have been applied to DNA accessibility related to chromatin and nucleosome occupancy (45). However, these techniques require a huge volume of data for training if they are to predict just a small fraction of data with marginally improved accuracy, as compared to more traditional approaches based on *k*-mers, and they have not shed new light on the sequence dependence of occupancy. Here we test the ability of a general set of measures, statistical and algorithmic, to be informative about nucleosome occupancy.

**Figure 4.**
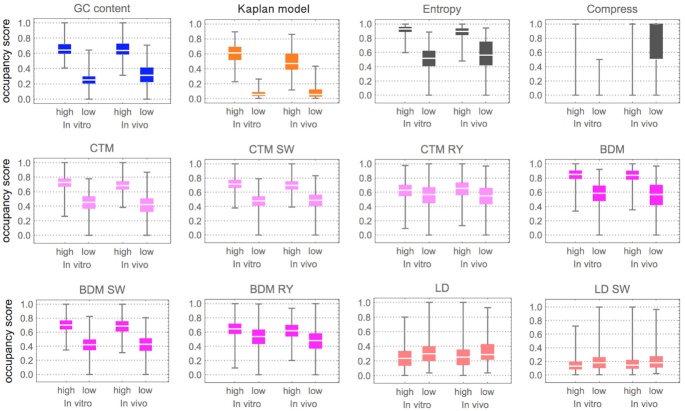
Box plots of informative indices for top highest and bottom lowest occupancies on Yeast chromosome 14 of 100K bp, representing about 1% of the Yeast genome. The occupancy score is given by a re-scaling function of the complexity value *f*_c_ (*y*-axis) where the highest score value is 1 and the lowest 0. In the case of the Kaplan model, *f*_c_ is the score calculated by the model (7) itself, which retrieves probability values between 0 and 1. Other cases not shown (e.g. entropy rate or Compress on RY or SW) yielded no significant results. Magenta and pink (bright colours) signify measures of algorithmic complexity; the information-theoretic based measures are in dark grey. This segment in chromosome 14 is a randomly chosen sequence for which both *in vivo* and *in vitro* nucleosomal positioning values are available, which is likely the reason it is frequently used in the literature. When integrating more regions the errors accumulated due to large gaps of missing values produced results impossible to compare, thereby forcing us to constrain the experiment to this segment.

Here, we have gone beyond previous attempts to connect and apply measures of complexity to structural and functional properties of genomic DNA, specifically in the highly active and open challenge of nucleosome occupancy in molecular biology. While more investigation is needed, these first experiments strongly suggest, and we report, that:

1. Algorithmic measures such as CTM and BDM of DNA sequences are informative of nucleosome occupancy. This is especially true for:
  - Sequences in which GC content may not be as informative. Because, unlike *k*-mer frequency-based scores, BDM does not trivially correlate with GC content, as shown in Figure 2. In contrast, we know that the correlation of the Kaplan model with GC content is very high (overall ∼0.90 Pearson correlation based on Figure 1).
  - Sequences with very high or very low nucleosome occupancy (Figure 4). These sequences have been reported to have particular biological significance (see (46)). While the difference between low and high complexity is still best captured by the Kaplan model, the entropy and complexity-based indices are also highly informative for high occupancy and complexity-based measures remain informative for occupancy.
2. Computational biologists can estimate CTM and BDM values for candidate nucleosomal DNA sequences of any length using an online complexity calculator, http://complexitycalculator.com and following these steps:
  - Chunk the DNA sequence into subsequences of desired sliding window length,
  - Introduce each DNA sequence into the calculator field (see Supplementary Figure S1 in the Sup. Mat.),
  - Retrieve the value for each query,
  - The ordered time series of CTM/BDM values is the score function. Lowest values are more likely to signal a nucleosome centre according to the results in this paper.

Source code to perform these calculations without querying the website is also available online, written in R and easily accessible through the acss package, fully documented at: https://cran.r-project.org/web/packages/acss/acss.pdf.

This suggests that the training-free CTM, BDM and LD-based indices may cover domains previously left uncovered by the Kaplan model, and that these new measures can complement current protocols, making it possible to combine these measures with the Kaplan method to produce even more accurate predictions. The results are actually be very timely because understanding nucleosome positioning (and chromatin state) is becoming a key factor in designing CRISPR/Cas9 gene editing experiments (47)

Further investigation of the indices’ application to high nucleosome occupancy should be performed by using some other standard organisms, such as *Caenorhabditis elegans*, on which *in vitro* and *in vivo* nucleosomal data is comprehensive. In the future, we will also explore applying these tools on sequences for high GC content but this is not in the scope of this current paper.

The algorithmic complexity approach is interesting when following our methods (as opposed to e.g. popular lossless compression algorithms) because they do not only provide scores by numerical estimations for different nucleosomal and genomic regions according to their function or structure but they also provide access to the actual models (computer programs) that are able to reproduce the complexity of each region and represent thus a set of candidate generative models for different regions with shared (low versus high) algorithmic properties. These models offer a window (as opposed to a black box) to further inspection of underlying causes for feature extraction and knowledge discovery in the form of, e.g. new longer range *k*-mers. The fact that our algorithmic indexes can account for structural properties and have informative power suggests that there are common mechanistic properties shared among similar structural genomic regions.

A direction for future research suggested by our work is therefore the exploration of the use of these complexity indices to complement current models and recent machine learning approaches for reducing the feature space, by, e.g., determining which *k*-mers are more and less informative and occur as a result of a recursive property according to the set of computer programs that reproduce a region.

Another direction to explore could involve an extensive investigation of the possible use of genomic profiling for other types of structural and functional properties of DNA according to their algorithmic complexity and algorithmic probability indices, with a view to contributing to, e.g., HiC techniques or protein encoding/promoter/enhancer region detection, and to furthering our understanding of the effect of extending the alphabet transformation of a sequence to epigenetics.

## DATA AVAILABILITY

The method was implemented as an additional Tab in the Complexity Calculator online: http://complexitycalculator.com/.

## Supplementary Material

gkz750_Supplemental_File
