# Supplementary material for: Training-free measures based on algorithmic probability identify high nucleosome occupancy in DNA sequences
Source: Nucleic Acids Res. 2019 Sep 12;47(20):e129. doi: 10.1093/nar/gkz750 (PMC6846163; doi:10.1093/nar/gkz750)
Supplement: gkz750_Supplemental_File [file gkz750_supplemental_file.pdf]

Supplementary Material *

1 Indices of Information and of Algorithmic Complexity

Here we describe alternative measures to explore correlations from an information-theoretic and algorithmic (hence causal) complexity perspective.

1.1 Shannon Entropy

Central to information theory is the concept of Shannon's entropy, which quantifies the average number of bits needed to store or communicate a message. Entropy determines that one cannot store (and therefore communicate) a message with n different symbols in less than $\log(n)$ bits. In this sense, Entropy determines a lower limit below which no message can be further compressed, not even in principle. Another application (or interpretation) of Shannon's information theory is as a measure for quantifying the *uncertainty* involved in predicting the value of a random variable.

Shannon defined the Entropy H of a discrete random variable X with possible values $x_1, \ldots, x_n$ and probability distribution $P(X)$ as:

$$H(X) = -\sum_{i=1}^{n} P(x_i) \log_2 P(x_i)$$

where if $P(x_i) = 0$ for some i, the value of the corresponding summand $0\ log_2(0)$ is taken to be 0.

1.1.1 Entropy Rate

The function R gives what is variously denominated as rate or block Entropy, and is Shannon Entropy over blocks or subsequences of X of length b. That is,

$$H_R(X) = \min_{b=1}^{b=|X|} H(X_b)$$

*Training-free Measures Based on Algorithmic Probability Identify High Nucleosome Occupancy in DNA Sequences, Zenil and Minary, Nucleic Acids Research, 2019

If the sequence is not statistically random, then $H_R(X)$ will reach a low value for some b, and if random, then it will be maximally entropic for all blocks b. $H_R(X)$ is computationally intractable as a function of sequence size, and typically upper bounds are realistically calculated for a fixed value of b (e.g. a window length). Notice that, as discussed in the main text, having maximal Entropy does not by any means imply algorithmic randomness (c.f. 1.3).

1.2 Lossless compression algorithms

Two widely used lossless compression algorithms were employed. On the one hand, Bzip2 is a lossless compression method that uses several layers of compression techniques stacked one on top of the other, including Run-length encoding (RLE), Burrows–Wheeler transform (BWT), Move to Front (MTF) transform, and Huffman coding, among other sequential transformations. Bzip2 compresses more effectively than LZW, LZ77 and Deflate, but is considerably slower.

On the other hand, *Compress* is a lossless compression algorithm based on the LZW compression algorithm. Lempel–Ziv–Welch (LZW) is a lossless data compression algorithm created by Abraham Lempel, Jacob Ziv, and Terry Welch, and is considered universal for an infinite sliding window (in practice the sliding window is bounded by memory or choice). It is considered *universal* in the sense of Shannon Entropy, meaning that it approximates the Entropy rate of the source (an input in the form of a file/sequence). It is the algorithm of the widely used Unix file compression utility 'Compress', and is currently in the international public domain.

1.3 Measures of Algorithmic Complexity

A binary sequence s is said to be random if its Kolmogorov-Chaitin complexity [7, 10, 4] $C(s)$ is at least twice its length. It is a measure of the computational resources needed to specify the object. Formally,

$$C(s) = \min\{|p| : T(p) = s\}$$

where p is a program that outputs s running on a universal Turing machine T. C as a function taking s to the length of the shortest computer program that produces s is semi-computable, and upper bound estimations are possible. The measure is today the accepted mathematical definition of randomness, among other reasons because it has been proven to be mathematically robust by virtue of the fact that several independent definitions converge to it.

The invariance theorem guarantees that complexity values will only diverge by a constant (e.g. the length of a compiler, a translation program between T_1 and T_2) and will converge at the limit. Formally,

$$|C(s)_{T_1} - C(s)_{T_2}| < c$$

1.3.1 Lossless Compression as Approximation to C

Lossless compression is traditionally the method of choice when a measure of algorithmic content related to Kolmogorov-Chaitin complexity C is needed. The Kolmogorov-Chaitin complexity of a sequence s is defined as the length of the shortest computer program p that outputs s running on a reference universal Turing machine T. While lossless compression is equivalent to algorithmic complexity, actual implementations of lossless compression (e.g. Compress) are heavily based upon Entropy rate estimations [13, 14] that mostly deal with statistical repetitions or k-mers of up to a window length size L, such that $k \leq L$.

1.3.2 Algorithmic Probability as Approximation to C

Another approach consists in making estimations by way of a related measure, *Algorithmic Probability* [6, 9]. The Algorithmic Probability of a sequence s is the probability that s is produced by a random computer program p when running on a reference Turing machine T. Both algorithmic complexity and Algorithmic Probability rely on T, but invariance theorems for both guarantee that the choice of T is asymptotically negligible.

One way to minimise the impact of the choice of T is to average across a large set of different Turing machines, all of the same size. The chief advantage of algorithmic indices is that causal signals in a sequence may escape entropic measures if they do not produce statistical regularities. And it has been the case that increasing the length of k in k-nucleotide models of structural properties of DNA has not returned more than a marginal advantage.

The Algorithmic Probability [10] (also known as Levin's semi-measure [8]) of a sequence s is a measure that describes the expected probability of a random program p running on a universal prefix-free Turing machine T producing s. Formally,

$$m(s) = \sum_{p:T(p)=s} 1/2^{|p|}$$

The Coding theorem beautifully connects $C(s)$ and $m(s)$:

$$C(s) \sim -\log m(s)$$

1.3.3 Bennett's Logical Depth

Another measure of great interest is *logical depth* [2]. The logical depth (LD) of a sequence s is the shortest time logged by the shortest programs p_i that produce s when running on a universal reference Turing machine. In other words, just as algorithmic complexity is associated with lossless compression, LD can be associated with the shortest time that a Turing machine takes to decompress the sequence s from its shortest computer description. A multiplicative invariance theorem for LD has also been proven [2]. Estimations of Algorithmic Probability and logical depth of DNA sequences were performed as determined in [6, 9].

Unlike algorithmic (Kolmogorov-Chaitin) complexity C, logical depth is a measure related to 'structure' rather than randomness. LD can be identified with biological complexity [3, 5] and is therefore of great interest when comparing different genomic regions.

1.4 Measures Based on Algorithmic Probability and on Logical Depth

The *Coding theorem method* (or simply CTM) is a method [6, 9] rooted in the relation between $C(s)$ and $m(s)$ specified by Algorithmic Probability [10, 8], that is, between frequency of production of a sequence from a random program and its Kolmogorov-Chaitin complexity as described by Algorithmic Probability. Essentially, it uses the fact that the more frequent a sequence the lower its Kolmogorov-Chaitin complexity, and sequences of lower frequency have higher Kolmogorov-Chaitin complexity. Unlike algorithms for lossless compression, the Algorithmic Probability approach not only produces estimations of C for sequences with statistical regularities, but it is deeply rooted in a computational model of Algorithmic Probability, and therefore, unlike lossless compression, has the potential to identify regularities that are not statistical (e.g. a sequence such as 1234...), that is, sequences with high Entropy or no statistical regularities but low algorithmic complexity [13, 12].

Let (n, m) be the space of all n-state m-symbol Turing machines, $n, m > 1$ and s a sequence, then:

$$D(n,m)(s) = \frac{|\{T \in (n,m) : T \textit{ produces } s\}|}{|\{T \in (n,m)\}|}$$

where T is a standard Turing machine as defined in the Busy Beaver problem by Radó [11] with 4 symbols (in preparation for the calculation of the DNA alphabet size).

Then, using the relation established by the Coding theorem, we have:

$$CTM(s) = -\log_2(D(n,m)(s))$$

That is, the more frequently a sequence is produced the lower its Kolmogorov-Chaitin complexity, and vice versa. CTM is an upper bound estimation of Kologorov-Chaitin complexity.

From CTM, a measure of Logical Depth can also be estimated–as the computing time that the shortest Turing machine (i.e. the first in the quasi-lexicographic order) takes to produce its output s before halting. CTM thus produces both an empirical distribution of sequences up to a certain size, and an LD estimation based on the same computational model.

Because CTM is computationally very expensive (equivalent to the Busy Beaver problem [11]), only short sequences (currently only up to length $k = 12$) have associated estimations of their algorithmic complexity. To approximate the complexity of genomic DNA sequences up to length $k = 12$, we calculated $D(5, 4)(s)$, from which $CTM(s)$ was approximated.

Table 1: Spearman correlation values of complexity score functions vs. the Wedge dinucleotide model prediction of DNA curvature on 20 synthetically generated DNA sequences depicted in Table 4

	**GC content**	**Entropy**	**Entropy rate** (4)	**Compress**	**BZip2**	**BDM**	**LD**
rho	-0.45	-0.44	-0.57	-0.58	-0.45	-0.57	0.65
p	0.047	0.051	0.0094	0.0079	0.048	0.0083	0.0019

To calculate the Algorithmic Probability of a DNA sequence (e.g. the sliding window of length 147 nt) we produced an empirical Algorithmic Probability distribution from $(5, 4)$ to compare with by running a sample of 325 433 427 739 Turing machines with up to 5 states and 4 symbols (the number of nucleotides in a DNA sequence) with empty input (as required by Algorithmic Probability). The resulting distribution came from 325 378 582 327 non-unique sequences (after removal of those sequences only produced by 5 or fewer machines/programs).

1.5 Relation of BDM to Shannon Entropy and GC Content

The Block Decomposition Method (BDM) is a divide-and-conquer method that can be applied to longer sequences on which local approximations of $C(s)$ using CTM can be averaged, thereby extending the range of application of CTM. Formally,

$$BDM(s, k) = \sum_{s_k} \log(n) + CTM(r)$$

where the set of subsequences s_k is composed of the pairs (r, n), where r is an element of the decomposition of sequence s of size k, and n the multiplicity of each subsequence of length k. $BDM(s)$ is a computable approximation from below to the algorithmic information complexity of s, $C(s)$. BDM approximations to C improve with smaller departures (i.e. longer k-mers) from the Coding Theorem method. When k decreases in size, however, we have shown [14] that BDM approximates the Shannon Entropy of s for the chosen k-mer distribution. In this sense, BDM is a hybrid complexity measure that in the 'worst case' behaves like Shannon Entropy, and in the best approximates C. We have also shown that BDM is robust when, instead of partitioning a sequence, overlapping subsequences are used, but this latter method tends to over-fit the value of the resultant complexity of the original sequence that was broken into k-mers.

Table 2: Distance in number of nucleotides to local minimum (local maximum for LD and greatest local min/max for GC content) around a window of length 73 nts. In all cases, the same sequence was used and was assembled by flanking true nucleosomal regions with pseudo-randomly generated sequences with the same GC content as the mean of the GC content of the nucleosomal regions. Even in cases when GC content is not informative (by design) because neither the local min or max values were found closer to the centres than 20 nts on average (and median of 22), max values of BDM were better able to pinpoint nucleosome centres in a large number of cases and with an accuracy of less than 10 nts on average (and a median of less than 7 nts). Entropy was found to be off by around 11.5 nts on average (median of 10 nts), lossless compression by more than 21 nts (median of 36), and LD (max values) by less than 7 nts on average (median 4.5 nts). Unlike Kaplan's, BDM and LD are informative but training-free, followed closely by entropy.

LD

601	603	605	5Sr DNA	pGub	chicken $\beta-$ globulin		
3	12	13	14	12	8		
**msat**	**CAG**	**TATA**	**CA**	**NoSecs**	**TGGA**	**TGA**	**BadSecs**
6	3	5	2	4	3	4	3

BDM

601	603	605	5Sr DNA	pGub	chicken $\beta-$ globulin		
15	11	19	22	15	6		
**msat**	**CAG**	**TATA**	**CA**	**NoSecs**	**TGGA**	**TGA**	**BadSecs**
8	1	2	0	29	1	2	6

Entropy

601	603	605	5Sr DNA	pGub	chicken $\beta-$ globulin		
15	2	14	12	10	8		
**msat**	**CAG**	**TATA**	**CA**	**NoSecs**	**TGGA**	**TGA**	**BadSecs**
17	22	3	31	8	6	4	

GC content

601	603	605	5Sr DNA	pGub	chicken $\beta-$ globulin		
32	25	36	25	15	25		
**msat**	**CAG**	**TATA**	**CA**	**NoSecs**	**TGGA**	**TGA**	**BadSecs**
16	32, 26	22	16	18	26		

Compression

601	603	605	5Sr DNA	pGub	chicken $\beta-$ globulin		
36	4	36	36	36	36		
**msat**	**CAG**	**TATA**	**CA**	**NoSecs**	**TGGA**	**TGA**	**BadSecs**
12	5	3	4	36	3	2	36

Table 3: 14 Experimental nucleosome sequences [1]. Only the first 6 have known dyads

name	dyad position	sequence
601	74	ACAGGATGTATATATCTGACACGTGCCTGGAGACTAGGGAGTA<br>ATCCCCTTGGCGGTTAAAACGCGGGGGACAGCGCGTACGTGCG<br>TTTAAGCGGTGCTAGAGCTGTCTACGACCAATTGAGCGGCCTCG<br>GCACCGGGATTCTCCAG
603	154	CGAGACATACACGAATATGGCGTTTTCCTAGTACAAATCACCCCA<br>GCGTGACGCGTAAAATAATCGACACTCTCGGGTGCCCAGTTCGC<br>GCGCCCACCTACCGTGTGAAGTCGTCACTCGGGCTTCTAAGTACG<br>CTTAGGCCACGGTAGAGGGCAATCCAAGGCTAACCACCGTGCAT<br>CGATGTTGAAAGAGGCCCTCCGTCCTTATTACTTCAAGTCCCTGG<br>GGTACCGTTTC
605	132	TACTGGTTGGTGTGACAGATGCTCTAGATGGCGATACTGACAGG<br>TCAAGGTTCGGACGACGCGGGATATGGGGTGCCTATCGCACATT<br>GAGTGCGAGACCGGTCTAGATACGCTTAAACGACGTTACAACCC<br>TAGCCCCGTCGTTTTAGCCGCCCAAGGGTATTCAAGCTCGACGCT<br>AATCACCTATTGAGCCGGTATCCACCGTCACGACCATATTAATAG<br>GACACGCCG
5Sr DNA	74, 92	AACGAATAACTTCCAGGGATTTATAAGCCGATGACGTCATAACAT<br>CCCTGACCCTTTAAATAGCTTAACTTTCATCAAGCAAGAGCCTAC<br>GACCATACCATGCTGAATATACCGGTTCTCGTCCGATCACCGAAG<br>TCAAGCAGCATAGGGCTCGGTTAGTACTTGGATGGGAGACCGCC<br>TGGGAATACCG
pGub	84, 104	GATCCTCTAGACGGAGGACAGTCCTCCGGTTACCTTCGAACCACGT<br>GGCCGTCTAGATGCTGACTCATTGTCGACACGCGTAGATCTGCTAG<br>CATCGATCCATGGACTAGTCTCGAGTTTAAAGATATCCAGCTGCCC<br>GGGAGGCCTTCGCGAAATATTGGTACCCCATGGAATCGAGGGATC
chicken β-globulin	125	CTGGTGTGCTGGGAGGAAGGACCCAACAGACCCAAGCTGTGGTC<br>TCCTGCCTCACAGCAATGCAGAGTGCTGTGGTTTGGAATGTGTGA<br>GGGGCACCCAGCCTGGCGCGCGCTGTGCTCACAGCACTGGGGTG<br>AGCACAGGGTGCCATGCCCACACCGTGCATGGGGATGTATGGCGC<br>ACTCCGGTATAGAGCTGCAGAGCTGGGAATCGGGGGG
mouse minor satellite		ATTTGTAGAACAGTGTATATCAATGAGCTACAATGAAAATCATGGA<br>AAATGATAAAAACCACACTGTAGAACATATTAGATGAGTGAGTTA<br>CACTGAAAAACACATCCGTTGGAAACCGGCAT
CAG		AGCAGCAGCAGCAACAGTAGTAGAAGCAGCAGCACTAACGACAG<br>CACAGCAGTAGCAGTAATAGAAGCAGCAGCAGCAGCAGTAGCAG<br>TAGCAGCAGCAGCAGCAGCAATTTCAACAACAGCAGCAGCAGCT
TATA		AGGTCTATAAGCGTCTATAAGCGTCTATGAACGTCTATAAACGTCT<br>ATAAACGCCTATAAACGCCTATAAACGCCTATACAAGCCTATAAAC<br>GCCTATACACGTCTATGCACGACTATACACGTCT
CA		GAGAGTAACACAGGCACAGGTGTGGAGAGTAACACAGGCACAG<br>GTGTGGGAGAGTGACACACAGGCACAGGTGAGGAGAGTACACA<br>CAGGCACAGGTGTGGAGAGCACACACAGGTGCGGAGAG
NoSecs		GGGCTGTAGAATCTGATGGAGGTGTAGGATGGATGGACAGTATGA<br>CAAAAGGGTACTAGCCTGGGACAGCAGGATTGGTGGAAAGGTTA<br>CAGGCAGGCCCAGCAGGCTCGGACGCTGTATAGAG
TGGA		AGATGGATGGATGATGGATGGATGATGGATAGATGGATGATGGAT<br>GGATGGATGATGATGGATGAATAGATGGATGGATGGATGATGGAT<br>GGATGGACGATGGATGGATAGATGGATGGATGG
TGA		ATAGATGGATGAGTGGATGGATGGGTGGATGGATAGATGGGTGG<br>ATGGGTGGATGGGTGGATGGATGATGGATGGATGAGTGGATGGA<br>TGGATGGATGGGTGGATGGGTGGACGG
BadSecs		TCTAGAGTGTACAACTATCTACCCTGTAGGCATCAAGTCTATTTCGG<br>TAATCACTGCAGTTGCATCATTTCGATACGTTGCTCTTGCTTCGCTAG<br>CAACGGACGATCGTACAAGCAC

Table 4: The 20 short DNA sequences artificially generated covering a wide range of patterns and regularities used to find informative measures of DNA curvature.

AAAAAAAAAAAA	ATATATATATAT	AAAAAATTTTTT
AAAAAAAAATAA	AAAAAAAACAAT	AAGATCTACACT
ATAGAACGCTCC	ACCTATGAAAGC	TAGGCGGCGGGC
TCGTTCGCGAAT	TGCACGTGTGGA	CTAAACACAATA
CTCTCAGGTCGT	CTCGTGGATATC	CCACGATCCCGT
GGCGGGGGGTGG	GGGGGGGCGGGC	GGGGGGCCCCCC
GCGCGCGCGCGC	GGGGGGGGGGGG	

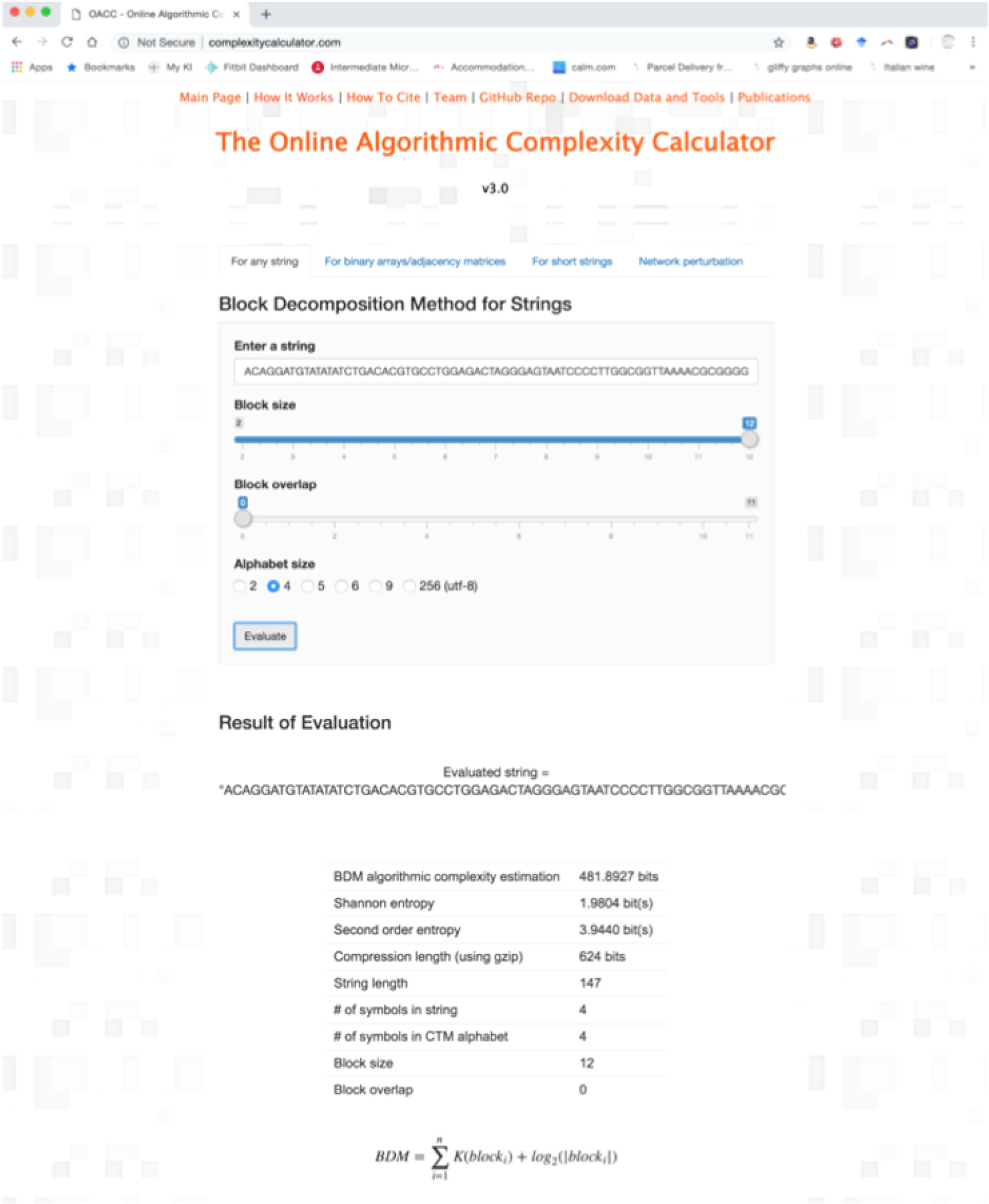

Figure 1: Evaluation of nucleosome 601 DNA sequence in the Online Algorithmic Complexity Calculator available free at `http://complexitycalculator.com/`. To reproduce scores between 0 and 1 as reported in all the results, it suffices to rescale all values between 0 and 1.

References

[1] van der Heijden T, van Vugt JJ, Logie C, van Noort J (2012) Sequence-based prediction of single nucleosome positioning and genome-wide nucleosome occupancy. *Proceedings of the National Academy of Sciences* 109(38):E2514–E2522.

[2] Bennett CH (1995) Logical depth and physical complexity. *The Universal Turing Machine, A Half-Century Survey* pp. 207–235.

[3] Bennett CH (1993) Dissipation, information, computational complexity and the definition of organisation in *Santa Fe Institute Studies in the Sciences of Complexity -Proceedings Volume-*. (Addison-Wesley Publishing Company), Vol. 1, pp. 215–215.

[4] Chaitin GJ (1969) On the length of programs for computing finite binary sequences: statistical considerations. *Journal of the ACM (JACM)* 16(1):145–159.

[5] Collier JD (1998) Information increase in biological systems: how does adaptation fit? in *Evolutionary systems*. (Springer), pp. 129–139.

[6] Delahaye JP, Zenil H (2012) Numerical evaluation of algorithmic complexity for short strings: A glance into the innermost structure of randomness. *Applied Mathematics and Computation* 219(1):63–77.

[7] Kolmogorov AN (1968) Three approaches to the quantitative definition of information. *International Journal of Computer Mathematics* 2(1-4):157–168.

[8] Levin LA (1974) Laws of information conservation (nongrowth) and aspects of the foundation of probability theory. *Problemy Peredachi Informatsii* 10(3):30–35.

[9] Soler-Toscano F, Zenil H, Delahaye JP, Gauvrit N (2014) Calculating Kolmogorov complexity from the output frequency distributions of small Turing machines. *PloS one* 9(5):e96223.

[10] Solomonoff RJ (1964) A formal theory of inductive inference. parts i and ii. *Information and control* 7(1):1–22 and 224–254.

[11] Rado T (1962) On non-computable functions. *Bell System Technical Journal* 41(3):877–884.

[12] Zenil H (2017) Algorithmic data analytics, small data matters and correlation versus causation in *Computability of the World? Philosophy and Science in the Age of Big Data*, eds. Pietsch W, Wernecke J, Ott M. Springer Verlag, pp 453-475.

[13] Zenil H, Badillo L, Hernández-Orozco, Hernández-Quiroz F (2018) Coding-theorem Like Behaviour and Emergence of the Universal Distribution from Resource-bounded Algorithmic Probability, *International Journal of Parallel Emergent and Distributed Systems.*

[14] Zenil H, Soler-Toscano F, Kiani NA, Hernández-Orozco S, Rueda-Toicen A (2018) A decomposition method for global evaluation of Shannon Entropy and local estimations of algorithmic complexity. *Entropy* 20(8), 605.
